# Comparing the acceptability of total diet replacement and food-based low-calorie diets for type 2 diabetes remission amongst Southeast Asians: a public and patient involvement activity

**DOI:** 10.3310/nihropenres.13233.1

**Published:** 2021-11-30

**Authors:** Grace Farhat, Sajda Majeed, Martin K. Rutter, Basil Issa, Michelle Harvie

**Affiliations:** 1 Health Professions, Manchester Metropolitan University, Manchester, M15 6BG, UK; 2 Patient and public engagement consultant, Patient and public engagement consultant, Burnley, UK; 3 Division of Endocrinology, Diabetes and Gastroenterology, Faculty of Biology, Medicine and Health, School of Medical Sciences, University of Manchester, Manchester, UK; 4 Manchester Diabetes centre, Endocrinology and Metabolism Centre, Manchester University NHS Foundation Trust, Manchester Academic Health Science Centre, Manchester, UK; 5 Department of Endocrinology, Manchester University Foundation Trust, Manchester, UK; 6 Prevent Breast Cancer Research Unit, Manchester University Hospital Foundation NHS Trust, Manchester, UK; 7 NIHR Manchester Biomedical Research Centre, NIHR, Manchester, UK; 8 UK Division of Cancer Sciences, University of Manchester, Manchester, Manchester, UK

**Keywords:** Type 2 diabetes; South East Asian population; diabetes remission; primary care; total diet replacement; weight control

## Abstract

**Background:**

With type 2 diabetes prevalence rising at alarming rates, low-calorie diets (total diet replacement and food-based low-calorie diets) are increasingly used to induce weight loss and achieve diabetes remission. The effectiveness of these diets has been primarily tested in the UK white population but not in the Southeast Asian population at high risk of diabetes. Obtaining the opinion of members of the community on what would constitute a culturally acceptable diet is essential for successful interventions aiming to achieve diabetes remission in the southeast Asian population.

**Methods:**

We organised two patient and public involvement activities in the North West of England to understand views of people from the Southeast Asian population on whether low-calorie diets (850 Kcal) in the form of total diet replacement or food-based meals, are acceptable dietary interventions to achieve diabetes remission.

**Results:**

Thirteen people, with either type 2 diabetes or having someone with diabetes in the family attended a virtual or a face-to-face meeting. Low-calorie total diet replacement in the form of soups and shakes was considered unacceptable, while there was a preference for a culturally tailored low-calorie food-based diet. Ready-made portion controlled catered meals were suggested as an excellent approach to improve adherence.

**Conclusions:**

This work provided valuable insights to shape a future study looking at the feasibility to reverse diabetes in primary care through dietary intervention in the Southeast Asian population.

## Introduction

Type 2 diabetes has gained significant research interest due to its emergence as a global epidemic, causing significant health and economic impacts ^1, 2^. Diabetes UK has been committed to tackling the diabetes crisis in the UK population, and has invested heavily in ground-breaking research looking to treat diabetes and reduce the burden on the NHS ^3^. Diabetes UK-funded primary-care based trials were the first to report that type 2 diabetes can be reversed through weight loss brought about through low-calorie diets (~850 Kcal) in the form of total diet replacement (TDR) ^4, 5^, and efforts are now made to provide isocaloric low-calorie (850 kcal) food-based alternatives in primary care ^6^.

These approaches have been shown to be effective primarily in the White population in UK studies, and were shown to achieve diabetes remission in a Middle Eastern population ^7^. However, their value has not been considered in the Southeast Asian population, the second largest ethnic group in the UK, who have significantly higher prevalence of diabetes compared to the White population ^8, 9^. Southeast Asians have been historically less successful in weight loss programmes compared to White individuals, with greater reluctance to lose weight and a lesser body dissatisfaction ^10, 11^. It has been speculated that the lack of consideration and knowledge of educators in ethnic-specific foods has been one of the barriers for success ^12^, especially in a population in which food constitutes an important social tradition, drawing on major socio-cultural differences and variances in dietary habits when compared to other ethnicities ^13^. Therefore, obtaining the opinion of members of this community on what would constitute a culturally acceptable diet plan could help design an effective low energy dietary intervention in type 2 diabetes.

The Southeast Asian population has been majorly underrepresented in large national diabetes studies, which has limited culturally appropriate evidence-based recommendations ^14^. The barriers and facilitators to participation in health and diabetes research within the Southeast Asian population have been described elsewhere ^15, 16^. It is therefore important to look at the suitability and barriers for success for low-calorie interventions as a means of inducing diabetes remission in this population.

Patient and public involvement are essential in the success of clinical interventions ^17^. We therefore organised two patient and public involvement activities in the North West of England on the 1 ^st^ and 2 ^nd^ of September 2021, with the aim of informing on several elements of diabetes dietary interventions: including choice of diet (TDR or food-based), acceptability of measurements tools used in the study (quality of life questionnaire, step counters, diet diary collection), as well as barriers and facilitators to participation and adherence.

## Methods

### Participants

Patients and family members were recruited face-to-face and by telephone through a GP practice and with the assistance of a community education representative with strong community links helping to spread the word within different sub-ethnic groups (Pakistani and Indian groups). Invitations included the researchers’ contact details and were sent out by email and “Whatsapp” application either by the researcher directly or through the community representative. Overall, 13 out of 18 people accepted the invitation. Inclusion criteria included men and women over 18 years of age from a Southeast Asian background who are either patients with type 2 diabetes or have someone with type 2 diabetes in the household. English and non-English speakers were invited to attend, and the community representative was available to help with the translation.

### Meeting information

Five people living with diabetes attended a virtual meeting (4 women and 1 man), and 8 women who either have diabetes or who live with people with type 2 diabetes in their household, attended a face-to-face meeting at the Ghausia community centre (Burnley, Lancashire, UK). The face-to-face meeting was to support gender representation in a community where gender segregation is an important barrier, and to obtain insights from women who mostly handle the cooking in the household ^16^. Additionally, the face-to-face meeting was aimed to overcome internet illiteracy which would normally hinder participation. Both meetings were facilitated by the researcher (GF, PhD, female) with the help of a community representative (SM) who have prior experience of leading meetings in the community and who joined both panels and helped overcome language barriers. The researcher had no prior links with the community and was presented to the panel as a University lecturer interested in diabetes research. Each meeting lasted for one hour. Participants were emailed information on the planned topics of discussion prior to the meetings and participants were provided with additional paper copies during the face-to-face meeting ( *Extended data* ^18^). This consisted of an example of a diet consisting of soups and shakes, a 3-day meal plan low-calorie food-based diet [they were provided with information to explain that the diet has Mediterranean components (olive oil, fruits and vegetables), which have been shown to have beneficial effects on the prevention and management of type 2 diabetes and cardiovascular disease], a quality-of-life questionnaire (EuroQol 5D questionnaire) and information on the use of step counters. We provided gift vouchers (£20) as an acknowledgment for their participation.

Questions asked during the meetings are listed in Table 1. Audio recordings were made of the meetings, and the researcher also took field notes.

**Table 1.** Questions asked during both meetings.

1- What are your views on the TDR and the food based diet? What would be your preference if you are to take part? 2- What changes to the diets would you suggest that would make it more suitable to you? 3- If your preference is for food-based diets, would you prefer sets of meal plans that you are required to follow? 4- What are your views on the quality of life questionnaire? 5- Would you wear step counters during the study? 6- What are your views on online applications for recording diet? 7- Have you taken part in studies before? which ones or why not? 8- What do you think are the barriers for people in your community/your family to follow low calorie diets? 9- How could we help you be more engaged in research? 10- How important is it for your family/community members to support you when following the programme? 11- Would you be interested in taking part in the study if it is funded?

### Data analysis

Interviews were first transcribed by the researcher (GF), and a detailed summary of all responses was produced. Relevant information was then retained and included in the report.

### Ethical considerations

As this is a patient and public involvement and engagement work, ethical approval was not required, as per NIHR guidelines. Participants provided written informed consent to participate in the work and for their data to be published anonymously.

## Results

Characteristics of attendants are presented in Table 2.

**Table 2.** Characteristics of people who took part in both activities.

	Number of people (n)
**Gender (F/M)**	n=12/n=1
***Age (years)*** *<40* *40–65* *>65*	n=1 n=9 n=3
***Disease status*** *Type 2 diabetes patients* *Family members/carers* *Type 2 diabetes patients & carer*	n=7 n=4 n=2
***Education*** *No formal qualifications/not schooled* *GCSE/ O-Level* *Degree Level*	n=7 n=3 n=3
***Profession*** *Unemployed/stay at home* *Carer* *Teacher* *Self-employed* *Retired*	n=4 n=2 n=2 n=2 n=3

### Views on the use of total diet replacement

Overall, 11 out of 13 people stated that TDR for 12 weeks was an unacceptable intervention. Older people (n=3) felt that they would be particularly unwilling to follow this type of diet, and their perception is that that solid foods must be included to have a fulfilling diet. They provided examples of their preference as stated below:

*“Soups and shakes could be a short-term fix (2 weeks or so) but not a diet that could be adopted for 3 months” - Participant 1-Female (40–65 years)*
*“Too long” - Participant 2-Female (40–65 years)*
*“Soups won’t fill you up” - Participant 3-Female (>65 years)*
*“A soup represents for us a food you have when you are ill”- Participant 4-Female (>65 years)*
*“Adding Chapati to soups would be more acceptable” - Participant 5-Male (40–65 years)*

### Low-calorie food-based diets are more acceptable

Panels were provided with an example of a 3-day meal plan low-calorie food-based diet. They were provided with information to explain that the diet has Mediterranean components (olive oil, fruits and vegetables), which have been shown to have beneficial effects on the prevention and management of type 2 diabetes and cardiovascular disease.

Eleven participants reported that the food-based diet would be more acceptable than TDR, but there was a unanimous opinion (n=13) that it would have to be culturally tailored to the Southeast Asian population. There was a strong message that the use of spices would be essential for acceptance of the intervention, as well as the inclusion of staple foods (chapati, rice etc..). For those born outside the UK (n=8), it was reported that it would be crucial that they adhere to a strict traditional diet, while Southeast Asians born in the UK were more willing to accept non-traditional foods. Below are some statements reported by the panels:

*“Spices are needed for flavour” -Participant 1 -Female (40–65 years)*
*“Add traditional foods especially chapati and rice” -*Participants 2 & 3-Females *(40–65 years)*
*“Set-up meal plans (e.g., 14 menus) are preferred” -All participants*
*“Add more vegetables that could be cooked with less oil” -Participant 3 -Female (40–65 years)*
*“Allow vegan/vegetarian options” Participant 4-Female (40–65 years)*
*“Olive and olive oil are acceptable” -All participants*
*“Consider teeth problems in older adults” -Participant 5-Female (>65 years).*

### Set-up catered meal plans are suggested as a convenient option

While discussing food-based diet preferences, two members of the panel went on to discuss the idea of providing ready-made portion controlled catered meals. The idea received enthusiasm from the whole cohort, and it was suggested that this would be an excellent way to improve adherence among people, educate them on portions/ingredients, and give them an idea about cooking methods for when they planned to prepare similar meals for themselves.

*“Meal plans will help me understand what ingredients and portions to use so I can then later on prepare food by myself” – Participant 1-Female (40–65 years)*

### Support: family and community

The facilitator asked whether the presence of family and community support would be essential for the success of the intervention. Panels stressed the importance of peer support in the weight loss and diabetes remission journey. This includes peer support group meetings within the community (n=1). Patients (n=2) also welcomed the idea of having family members attending appointments and helping overcome language barriers. However, it was mentioned that “some meanings could be lost in the translation” (n=4), thus a translator with more expertise could be of greater help in conveying accurate information to patients. Another participant mentioned the potential importance of peer support group meetings in achieving adherence.

### Other components of the intervention

All other outcome measures in the intervention (step counters) as well as quality of life questionnaire were deemed acceptable (n=13), but only after the community representative explained their use to both panels. However, reporting diet through a phone app was reported to be unsuitable by 11 people. Therefore, using a paper record was preferred by the majority.

### Taking part in diabetes research studies

Participants expressed their enthusiasm in taking part in the study should it be funded. Five patients were very keen to follow an intervention that could achieve remission. Importantly, one participant stated that diabetes was not perceived as a major risk that requires action due to it being very common among their community. Participants (n=13) unanimously stated that they had not taken part in research studies before because they have never been asked. This statement contrasts with the findings of previous reports showing a lack of interest of this community in taking part in research studies ^9^. Widening recruitment strategies might be an important point to consider in future research.

## Discussion

### Strengths and limitations

This report has several strengths. To our knowledge, this is the first activity comparing the potential acceptability of TDR and food-based low-calorie diets for diabetes remission in a Southeast Asian population. In addition to a virtual meeting, we used face-to-face meetings to overcome internet illiteracy.

There are some limitations. Whilst attempts were made to ensure that the study cohort was representative of the background population, the small number of participants and our recruitment methods could impact the conclusions drawn from these meetings. The predominance of women in this activity, might limit the generalisability of these insights in male South east Asian population groups.

### Clinical and research implications

This work will help us design a randomised controlled study using low calorie diets in Southeast Asian people with type 2 diabetes with the aim of inducing remission. Based on these insights, we plan to avoid using TDR as an intervention for diabetes remission in the South east Asian population. Instead, this work suggests the potential utility of a food-based low-calorie intervention, including looking at the feasibility of administering catered meals in primary care when compared to usual care. Meal plans will be prepared together with members of the community and patient support members. Through the Greater Manchester Strategic Clinical Network and the Research for the Future intervention, we will promote engagement with this research for people with diabetes from the Southeast Asian population.

## Conclusions

The Southeast Asian population is an important target group for interventions designed to prevent or treat type 2 diabetes. This activity provided useful insights to shape future study looking at the feasibility to reverse diabetes in primary care through dietary intervention in a high-risk population, and to encourage more patients to become involved in diabetes research, leading in the long-term to improved quality of life, health, and economic benefits.

## Data availability

### Underlying data

Data collected was in form of notes and recordings. Participants were informed that all recordings would be discarded after the interview. Therefore, the underlying data for this research is not available. Data collected was qualitative and the article encompasses most of the recorded data in order to help inform future research.

### Extended data

Zenodo: Comparing the acceptability of total diet replacement and food-based low-calorie diets for type 2 diabetes remission amongst Southeast Asians: a public and patient involvement activity, https://doi.org/10.5281/zenodo.5720754 ^18^.

This project contains the information sheet that participants were provided with before and during the meetings.

### Reporting guidelines

Zenodo: COREQ checklist for ‘Comparing the acceptability of total diet replacement and food-based low-calorie diets for type 2 diabetes remission amongst Southeast Asians: a public and patient involvement activity’, https://doi.org/10.5281/zenodo.5720754 ^18^.

Data are available under the terms of the Creative Commons Attribution 4.0 International license (CC-BY 4.0).
