# Comparing the acceptability of total diet replacement and food-based low energy diets for type 2 diabetes remission amongst South Asians: a public and patient involvement activity

**DOI:** 10.3310/nihropenres.13233.4

**Published:** 2022-11-15

**Authors:** Grace Farhat, Sajda Majeed, Martin K. Rutter, Basil Issa, Michelle Harvie

**Affiliations:** 1 Department of Health Professions, Manchester Metropolitan University, Manchester, M15 6BG, UK; 2 Patient and public engagement consultant, Patient and public engagement consultant, Burnley, UK; 3 Division of Endocrinology, Diabetes and Gastroenterology, Faculty of Biology, Medicine and Health, School of Medical Sciences, University of Manchester, Manchester, UK; 4 Manchester Diabetes centre, Endocrinology and Metabolism Centre, Manchester University NHS Foundation Trust, Manchester Academic Health Science Centre, Manchester, UK; 5 Department of Endocrinology, Manchester University Foundation Trust, Manchester, UK; 6 Prevent Breast Cancer Research Unit, Manchester University Hospital Foundation NHS Trust, Manchester, UK; 7 NIHR Manchester Biomedical Research Centre, NIHR, Manchester, UK; 8 UK Division of Cancer Sciences, University of Manchester, Manchester, Manchester, UK

**Keywords:** Type 2 diabetes, South East Asian population, diabetes remission, primary care, total diet replacement, weight control

## Abstract

**Background:**

With type 2 diabetes prevalence rising, low energy diets (total diet replacement and food-based low energy diets) are increasingly used to induce weight loss and achieve diabetes remission. The effectiveness of these diets has been primarily tested in the UK white population but not in the south Asian population at high risk of diabetes. Obtaining the opinion of members of the community on what would constitute a culturally acceptable diet is essential for successful interventions aiming to achieve diabetes remission in south Asians.

**Methods:**

We organised two patient and public involvement activities in the North West of England to understand views of people from the south Asian population on whether low energy diets (850 Kcal) in the form of total diet replacement or food-based meals, are acceptable dietary interventions to achieve type 2 diabetes remission.

**Results:**

Thirteen people, with either type 2 diabetes or having someone with diabetes in the family attended a virtual or a face-to-face meeting. Low energy total diet replacement in the form of soups and shakes was considered unacceptable, while there was a preference for a culturally tailored low energy food-based diet. Ready-made portion controlled catered meals were suggested as a likely approach to improve adherence.

**Conclusions:**

This work provided valuable insights to shape a future study looking at the feasibility of a catered meal low-energy dietary intervention to induce T2D remission in primary care within the south Asian population.

## Introduction

Type 2 diabetes (T2D) rates are increasing worldwide causing significant health and economic impacts ^1, 2^. It is estimated that 4 million people (6% of the population) in the UK have T2D ^1^. Diabetes UK has been committed to address the increased diabetes prevalence in the UK population, and has invested heavily in ground-breaking research looking to treat T2D and reduce the pressure on the NHS ^3^. Diabetes UK-funded primary-care based trials were the first to report that T2D can be put in remission through weight loss brought about through low energy diets (~850 Kcal) in the form of total diet replacement (TDR) ^4, 5^, and efforts are now made to provide low energy (850 kcal) food-based alternatives in primary care ^6^.

These approaches have been shown to be effective primarily in the white population in UK studies, and similar rates of weight loss have been shown to achieve T2D diabetes remission in a Middle Eastern population ^7^ and in small populations of south Asians living in India ^8, 9^. However, their value has largely not been considered in the south Asian population, the second largest ethnic group in the UK, who have significantly higher prevalence of T2D diabetes compared to the white population ^10, 11^. South Asians have been historically less successful in weight loss programmes compared to white individuals, with greater reluctance to lose weight and a lesser body dissatisfaction ^12, 13^. The lack of consideration and knowledge of ethnic-specific foods amongst educators has been suggested as a barrier for success ^14^ in this population for whom food constitutes an important social tradition, drawing on major socio-cultural differences and variances in dietary habits when compared to other ethnicities ^15^. Therefore, obtaining the opinion of members of this community on what would constitute a culturally acceptable diet plan could help design an effective low energy dietary intervention in type 2 diabetes.

The south Asian population has been majorly underrepresented in large national diabetes studies, which has limited culturally appropriate evidence-based recommendations ^16^. The barriers and facilitators to participation in health and T2D diabetes research within the south Asian population (such as perceived participation to improve health, cultural and language barriers, and lack of interest) have been described elsewhere ^17^. It is therefore important to look at the suitability and barriers for success for low-energy interventions as a means of inducing T2D remission in this population.

Patient and public involvement refers to actively including service users and communities in designing and carrying out research, leading to a better success in clinical interventions ^18^. We therefore organised two patient and public involvement activities in the NorthWest of England on the 1 ^st^ and 2 ^nd^ of September 2021, with the aim of informing on several elements of T2D diabetes dietary interventions, including choice of diet (TDR or food-based), acceptability of measurements tools used in the study (quality of life questionnaire, step counters, diet diary collection) and barriers and facilitators to participation and adherence.

## Methods

### Participants

Patients and family members were recruited face-to-face and by telephone through a GP practice and with the assistance of a community education representative with strong community links helping to spread the word within different sub-ethnic populations (Pakistani, Bangladeshi and Indian groups) in community local groups. Invitations included the researchers’ contact details and were sent out by email and “Whatsapp” application either by the researcher directly or through the community representative. Overall, 18 people were approached, and 13 people accepted the invitation. Inclusion criteria included men and women over 18 years of age from a south Asian background who are either patients with type 2 diabetes or have someone with type 2 diabetes in the household. English and non-English speakers were invited to attend, and the community representative was available to help with the translation.

### Meeting information

Meetings were held at the planning stage of the study protocol. Five people living with diabetes attended a virtual meeting (4 women and 1 man), and 8 women who either have diabetes or who live with people with type 2 diabetes in their household, attended a face-to-face meeting at the Ghausia community centre (Burnley, Lancashire, UK). The face-to-face meeting was to support gender representation in a community where gender segregation is an important barrier ^19^. Additionally, the face-to-face meeting was aimed to overcome internet illiteracy which would normally hinder participation. Both meetings were facilitated by the researcher (GF, PhD, female) with the help of a community representative (SM) who have prior experience of leading meetings in the community and who joined both panels and helped overcome language barriers. The researcher had no prior links with the community and was presented to the panel as a University lecturer interested in diabetes research. Each meeting lasted for one hour. Participants were emailed information on the planned topics of discussion prior to the meetings and were provided with additional paper copies during the face-to-face meeting ( *Extended data* ^20^). The information pack comprised an example of a diet consisting of soups and shakes to be consumed for 12 weeks, a 3-day low energy food-based diet plan [they were provided with information to explain that the diet has Mediterranean components (olive oil, fruits and vegetables) which have beneficial effects on remission of T2D and cardiovascular health] ^21, 22^ as well as information on the use of step counters. We provided gift vouchers (£20) as an acknowledgment for volunteers’ participation.

Questions asked during the meetings are listed in Table 1. Audio recordings were made of the meetings, and the researcher also took field notes.

**Table 1.** Questions asked during both meetings.

1- What are your views on the TDR and the food-based diet? What would be your preference if you are to take part? 2- What changes to the diets would you suggest that would make it more suitable to you? 3- If your preference is for food-based diets, would you prefer sets of meal plans that you are required to follow? 4- What are your views on the quality-of-life questionnaire? 5- Would you wear step counters during the study? 6- What are your views on online applications for recording diet? 7- Have you taken part in studies before? which ones or why not? 8- What do you think are the barriers for people in your community/your family to follow low energy diets? 9- How could we help you be more engaged in research? 10- How important is it for your family/community members to support you when following the programme? 11- Would you be interested in taking part in the study if it is funded?

### Data analysis

Interviews were first transcribed verbatim by the researcher (GF), and a detailed summary of all responses was then produced. This summary was reviewed by the community representative. Relevant information was retained and included in the report.

### Ethical considerations

As this is a patient and public involvement and engagement work, ethical approval was not required, as per NIHR guidelines. Participants provided written informed consent to participate in the work and for their statements to be published anonymously.

## Results

Characteristics of attendants are presented in Table 2.

**Table 2.** Characteristics of people who took part in both activities.

	Number of people (n)
**Gender (F/M)**	n=12/n=1
***Age (years)*** *<40* *40–65* *>65*	n=1 n=9 n=3
***Sub-ethnicity*** *Pakistani* *Banghladeshi* *Indian* Pashtun	n=8 n=2 n=2 n=1
***Disease status*** *Type 2 diabetes patients* *Family members/carers* *Type 2 diabetes patients & carer*	n=7 n=4 n=2
***Education*** *No formal qualifications/not schooled* *GCSE/ O-Level* *Degree Level*	n=7 n=3 n=3
***Profession*** *Unemployed/stay at home* *Carer* *Teacher* *Self-employed* *Retired*	n=4 n=2 n=2 n=2 n=3
Socio economic status (IMD quintile *) *1 ^st^ quintile* *2 ^nd^ quintile* *3 ^rd^ quintile*	n=10 n=1 n=2

*IMD: Index of multiple deprivation score which is based on UK postcodes where 1 ^st^ quintile represents the most deprived areas, and 10 ^th^ quintile represents the least deprived ones. Source: UK data service ^23^.

### Views on the use of total diet replacement

Overall, 11 out of 13 people stated that TDR for 12 weeks was an unacceptable intervention. Older people (n=3) felt that they would be particularly unwilling to follow this type of diet, and their perception is that solid foods must be included to have a fulfilling diet. They provided examples of their preferences, as stated below:

*“Soups and shakes could be a short-term fix (2 weeks or so) but not a diet that could be adopted for 3 months” - Participant 1-Female (40–65 years)*
*“Too long” - Participant 2-Female (40–65 years)*
*“Soups won’t fill you up” - Participant 3-Female (>65 years)*
*“A soup represents for us a food you have when you are ill”- Participant 4-Female (>65 years)*
*“Adding Chapati to soups would be more acceptable” - Participant 5-Male (40–65 years)*

### Low energy food-based diets are more acceptable

Panels were provided with an example of a 3-day meal plan low energy food-based diet. They were provided with information to explain that the diet has Mediterranean components (olive oil, fruits and vegetables), which have been shown to have beneficial effects on the prevention and management of type 2 diabetes and cardiovascular disease.

Eleven participants reported that the food-based diet would be more acceptable than TDR, but there was a unanimous opinion (n=13) that it would have to be culturally tailored to the south Asian population. There was a strong message that the use of spices is essential for acceptance of the intervention, as well as the inclusion of staple foods (chapati, rice etc..). For those born outside the UK (n=8), it was reported that it would be crucial that they adhere to a strict traditional diet as this is linked to their home culture, while south Asians born in the UK were more willing to accept non-traditional foods. Below are some statements reported by the panels:

*“Spices are needed for flavour” -Participant 1 -Female (40–65 years)*
*“Add traditional foods especially chapati and rice” -*Participants 2 & 3-Females *(40–65 years)*
*“Set-up meal plans (e.g., 14 menus) are preferred” -All participants*
*“Add more vegetables that could be cooked with less oil” -Participant 3 -Female (40–65 years)*
*“Allow vegan/vegetarian options” Participant 4-Female (40–65 years)*
*“Olive and olive oil are acceptable” -All participants*
*“Consider teeth problems in older adults” -Participant 5-Female (>65 years).*

Although including a Mediterranean component in the food-based diet (together with its potential beneficial effects) might have made the food-based diet appear more positive, this particular element was not the subject of discussion in both activities. All panel discussions focused on the culturally appropriate elements in the food-based diet such as spices and traditional foods) that made it more appealing.

### Set-up catered meal plans are suggested as a convenient option

While discussing food-based diet preferences, two members of the panel went on to discuss the idea of providing ready-made portion controlled catered meals. The idea received enthusiasm from the whole group, and it was suggested that this would be an excellent way to improve adherence among people, educate them on portions/ingredients, and give them an idea about cooking methods for when they planned to prepare similar meals for themselves.

*“Meal plans will help me understand what ingredients and portions to use so I can then later on prepare food by myself” – Participant 1-Female (40–65 years)*

### Support: family and community

The facilitator asked whether the presence of family and community support would be essential for the success of the intervention. Panels stressed the importance of peer support in the weight loss and diabetes remission journey. This includes peer support group meetings within the community (n=1). Patients (n=2) also welcomed the idea of having family members attending appointments and helping overcome language barriers. However, it was mentioned that “some meanings could be lost in the translation” (n=4), thus a translator with more expertise could be of greater help in conveying accurate information to patients. Another participant mentioned the potential importance of peer support group meetings in achieving adherence.

### Other components of the intervention

Other outcome measures such as the use of step counters was deemed acceptable (n=13), but only after the community representative explained their use to both panels. However, reporting diet through a phone app was reported to be unsuitable by 11 people. Therefore, using a paper record was preferred by the majority.

### Taking part in diabetes research studies

Participants expressed their enthusiasm in taking part in the study should it be funded. Five patients were very keen to follow an intervention that could achieve remission. Importantly, one participant stated that diabetes was not perceived as a major risk that requires action due to it being very common among their community. Participants (n=13) unanimously stated that they had not taken part in research studies before because they have never been approached. This statement is in line with the findings of a previous report showing that people from this population did not participate in research studies because they have never been asked ^17^ Widening recruitment strategies is an important point to consider in future research.

## Discussion

### Strengths and limitations

This report has several strengths. To our knowledge, this is the first activity that gauges the opinion of individuals from the south Asian population regarding the acceptability of TDR or food-based low-energy diets and empower them to participate in future culturally tailored interventions to induce T2D remission. In addition to a virtual meeting, we used face-to-face meetings to overcome internet illiteracy. The presence of a community representative helped overcome language barriers and gain insights from both English and non-English speakers. We aimed to report the activities based on the international consensus for reporting PPIE activities in health and social science research (GRIPP2 guidelines) ( *Extended data* ^20^), which aim is to improve the quality and consistency of reporting patient and public involvement in research ^24^.

There are some limitations. Whilst attempts were made to ensure that the group of people was representative of the background population, the small number of participants and our recruitment methods could have impacted the conclusions drawn from these meetings. The predominance of women, people from Pakistani/Bangladeshi background and those from low socio-economic groups in this activity might have limited the generalisability of these insights in males, other south Asian population subgroups and people from higher socio-economic backgrounds. However, these activities were helpful in gathering insights from underrepresented and more traditional south Asian groups. Information could have benefited by being reviewed by more than one researcher to reduce potential researcher bias. There may also have been social desirability bias amongst the PPIE group. Additionally, our description of the potential health benefits of a Mediterranean diet may have positively impacted how participants viewed the food-based diet. Lastly, the lack of knowledge and use of TDR might have affected their acceptability. White individuals have previously expressed negative perceptions of TDR too, yet their opinions changed after use ^25^. Future research will be able to identify whether this will be the case in the south Asian population.

### Clinical and research implications

The community representative has been recruited to be part of the research team and was costed in the grant application as a “community delivery partner”. They will assist in facilitating group panel meetings throughout the study and planning dissemination activities, either directly or through the recruitment of a public contributor belonging to the same community.

Information from this activity including participants from a more traditional south Asian sub-group, suggest that TDR may have limited acceptability in this patient population. This work was pivotal in making changes to the project proposal, including study arms (we have reconsidered the addition of a TDR arm), outcome measures and dissemination tools. The potential utility of a food-based low-energy intervention was suggested, including looking at the feasibility of administering catered meals in primary care. Catered meal plans will be prepared together with members of the community and patient support members. An education element to increase knowledge of T2D risk and healthy eating was considered. This research for people with diabetes from the south Asian population will be promoted through the Greater Manchester Strategic Clinical Network and the Research for the Future campaign. This activity could potentially have economic benefits in terms of developing future interventions tailored to this population. As for future PPIE planning activities, more efforts into considering language barriers and cultural differences will improve the usefulness of this information and improve future research.

## Conclusions

The south Asian population is an important target group for interventions designed to induce T2D remission. This activity does not negate the use of TDR in this population but provided useful insights to shape a future study looking at the feasibility of food-based interventions for T2D remission in primary care in a high-risk population. This work aims to encourage more patients to become involved in T2D research, which may lead in the long-term to improved quality of life, health, and economic benefits.

## Data Availability

Information collected was in form of notes and recordings. Participants were informed that all recordings would be discarded after the interview. Therefore, the underlying information for this research is not available. Information collected was qualitative and the article encompasses most of the recorded information in order to help inform future research. Zenodo: Comparing the acceptability of total diet replacement and food-based low energy diets for type 2 diabetes remission amongst south Asians: a public and patient involvement activity, https://doi.org/10.5281/zenodo.5720754 ^20^. This project contains the information sheet that participants were provided with before and during the meetings. Zenodo: COREQ checklist for ‘Comparing the acceptability of total diet replacement and food-based low energy diets for type 2 diabetes remission amongst south Asians: a public and patient involvement activity’, https://doi.org/10.5281/zenodo.5720754 ^20^. Data are available under the terms of the Creative Commons Attribution 4.0 International license (CC-BY 4.0).
