# Supplementary material for: Comparing the acceptability of total diet replacement and food-based low energy diets for type 2 diabetes remission amongst South Asians: a public and patient involvement activity
Source: NIHR Open Res. 2022 Nov 15;1:24. Originally published 2021 Nov 30. [Version 4] doi: 10.3310/nihropenres.13233.4 (PMC10593247; doi:10.3310/nihropenres.13233.4)
Supplement: Peer review history

# Reviewer report: Reviewer response for version 4

**DOI:** 10.3310/nihropenres.14467.r28851

**Published:** 2022-11-16

**Authors:** Hanno Pijl

**Affiliations:** 1 Department of Internal Medicine, Leiden University Medical Center (LUMC), Leiden, The Netherlands

I still do not believe the paper contributes enough to be published. Although I acknowledge the importance of tailoring any (lifestyle) intervention to the needs of specific populations, the main issue with this work is: does it help us in deciding which dietary intervention is preferred by people of South Asian decent with T2D? The answer is a definitive no as far as I am concerned, given the very small number of representative participants and the various other issues I raised earlier.

Is the work clearly and accurately presented and does it cite the current literature?

Yes

If applicable, is the statistical analysis and its interpretation appropriate?

Not applicable

Are all the source data underlying the results available to ensure full reproducibility?

No

Is the study design appropriate and is the work technically sound?

No

Are the conclusions drawn adequately supported by the results?

No

Are sufficient details of methods and analysis provided to allow replication by others?

Yes

Reviewer Expertise:

Lifestyle treatment of diabetes and cancer

I confirm that I have read this submission and believe that I have an appropriate level of expertise to state that I do not consider it to be of an acceptable scientific standard, for reasons outlined above.

# Reviewer report: Reviewer response for version 4

**DOI:** 10.3310/nihropenres.14467.r28850

**Published:** 2022-11-16

**Authors:** Julie Abayomi

**Affiliations:** 1 School of Medicine and Nutrition, Faculty of Health, Social Care and Medicine, Edge Hill University, Ormskirk, UK

I am happy with the amendments that have been made to the paper.

Is the work clearly and accurately presented and does it cite the current literature?

Yes

If applicable, is the statistical analysis and its interpretation appropriate?

Not applicable

Are all the source data underlying the results available to ensure full reproducibility?

Yes

Is the study design appropriate and is the work technically sound?

Yes

Are the conclusions drawn adequately supported by the results?

Yes

Are sufficient details of methods and analysis provided to allow replication by others?

Yes

Reviewer Expertise:

Weight management and nutrition in pregnancy. Diabetes in pregnancy. PPI.

I confirm that I have read this submission and believe that I have an appropriate level of expertise to confirm that it is of an acceptable scientific standard.

# Reviewer report: Reviewer response for version 3

**DOI:** 10.3310/nihropenres.14396.r28769

**Published:** 2022-10-18

**Authors:** Julie Abayomi

**Affiliations:** 1 School of Medicine and Nutrition, Faculty of Health, Social Care and Medicine, Edge Hill University, Ormskirk, UK

Thank you for asking me to review this interesting and well written paper. The research is timely and important as achieving T2DM remission in the south Asian population is much needed. It is interesting to read that participants rejected the idea of meal replacements (soups and shakes) but were open to the idea of low calorie diets that included real, but culturally appropriate food. These findings will certainly help to influence culturally appropriate interventions for T2DM remission in the South Asian population in future.

 I have only a couple of suggestions to make and they both relate to the PPI aspects of the paper:

1. There are now international guidelines published, regarding the reporting of PPI, see GRIPP2 (Staniszewska *et al*., 2017 ^1^). It would be helpful for this paper to refer to these guidelines and show how the methods and findings relate to GRIPP2 guidelines.
2. To ensure that the main follow up study has adequate PPI throughout (from research design to dissemination of findings), did you use this initial work to recruit PPI representatives, to be part of the research team and costed in the grant application for the main study? Could the community representative be considered as a PPI rep for this present study? If so this would follow GRIPP2 guidelines and they should be named as a co-researcher and Co-author on the paper.

Is the work clearly and accurately presented and does it cite the current literature?

Yes

If applicable, is the statistical analysis and its interpretation appropriate?

Not applicable

Are all the source data underlying the results available to ensure full reproducibility?

Yes

Is the study design appropriate and is the work technically sound?

Yes

Are the conclusions drawn adequately supported by the results?

Yes

Are sufficient details of methods and analysis provided to allow replication by others?

Yes

Reviewer Expertise:

Weight management and nutrition in pregnancy. Diabetes in pregnancy. PPI.

I confirm that I have read this submission and believe that I have an appropriate level of expertise to confirm that it is of an acceptable scientific standard, however I have significant reservations, as outlined above.

## References

[1] : GRIPP2 reporting checklists: tools to improve reporting of patient and public involvement in research. *BMJ* .2017;358: 10.1136/bmj.j3453 j3453 10.1136/bmj.j3453 28768629PMC5539518

# Response

**Published:** 2022-11-01

**Authors:** Grace Farhat

**Affiliations:** Manchester Metropolitan University, UK

Many thanks for reviewing our manuscript. We are very grateful for providing helpful comments. Here, we respond to these point-by-point.

- There are now international guidelines published, regarding the reporting of PPI, see GRIPP2 (Staniszewska *et al*., 2017 ^1^ ). It would be helpful for this paper to refer to these guidelines and show how the methods and findings relate to GRIPP2 guidelines.

**Response 1: Thank you for referring to the guidelines. In response to this helpful comment, the report has been revised to include applicable elements of the GRIPP2 checklist (highlighted in the document). The checklist has also been added to the *extended data* ^20^.**

**The following statement has now been added to the report:**

*"We aimed to report the activities based on the international consensus for reporting PPIE activities in health and social science research (GRIPP2 guidelines) ( *Extended data* ^20^ ), which aim is to improve the quality and consistency of reporting patient and public involvement in research* **^24^" (** **Staniszewska et al, 2017).**

- To ensure that the main follow up study has adequate PPI throughout (from research design to dissemination of findings), did you use this initial work to recruit PPI representatives, to be part of the research team and costed in the grant application for the main study? Could the community representative be considered as a PPI rep for this present study? If so this would follow GRIPP2 guidelines and they should be named as a co-researcher and Co-author on the paper.

**Response 2: The community representative has become part of the research team and is a co-author on this paper (Sajda Majeed, MBE). They have also been costed in the grant application as a “community delivery partner” and will be able to help in developing and carrying out the study. As suggested by the reviewer, it is possible that the community representative will become our PPI representative. Otherwise, they will recruit a public contributor from the same community who will assist in** **facilitating PPIE advisory group panel meetings and planning dissemination activities. In response to this comment, this information has now been added to the *clinical and research implications* section of the report which reads,**

**“** *The community representative has been recruited to be part of the research team and was costed in the grant application as a “community delivery partner. They will assist in facilitating group panel meetings throughout the study and planning dissemination activities, either directly or through the recruitment of a public contributor belonging to the same community*.”

**Note: Because of the lack of involvement of members of this community in previous research, a PPIE representative of this community will be supported by a PPIE advisor who will provide the training required.**

# Reviewer report: Reviewer response for version 3

**DOI:** 10.3310/nihropenres.14396.r28585

**Published:** 2022-04-27

**Authors:** Hanno Pijl

**Affiliations:** 1 Department of Internal Medicine, Leiden University Medical Center (LUMC), Leiden, The Netherlands

Although the authors have adapted several points I raised appropriately, I am sorry to say that I do not think that a report of opinions of 13 people on one specific meal replacement strategy ("soups and shakes") for one specific period of time (12 weeks) has sufficient general meaning for clinical practice to be worth indexing.

Is the work clearly and accurately presented and does it cite the current literature?

Yes

If applicable, is the statistical analysis and its interpretation appropriate?

Not applicable

Are all the source data underlying the results available to ensure full reproducibility?

No

Is the study design appropriate and is the work technically sound?

No

Are the conclusions drawn adequately supported by the results?

No

Are sufficient details of methods and analysis provided to allow replication by others?

Yes

Reviewer Expertise:

Lifestyle treatment of diabetes and cancer

I confirm that I have read this submission and believe that I have an appropriate level of expertise to state that I do not consider it to be of an acceptable scientific standard, for reasons outlined above.

# Response

**Published:** 2022-05-26

**Authors:** Grace Farhat

**Affiliations:** Manchester Metropolitan University, UK

Thank you for your response. We have indeed attempted to address all comments you have kindly raised in the previous report. It is important to mention, however, that a PPIE activity is, by no means meant to have a general meaning for clinical practice. It is a preliminary activity that will help plan future interventions with an end point being to help clinical practice (https://arcem.nihr.ac.uk/sites/default/files/field/attachment/ARC%20EM_PPI%20Guidence.pdf). Whilst it is generally understood that PPIE has some flaws for not being a powered research study and ours have inherent limitations, we have indicated why this activity has been valuable in obtaining insights that will help planning future interventions (e.g targeting more traditional societies, using a community representative, considering language barriers) and ultimately help in improving practice in a minority ethnic group.

# Reviewer report: Reviewer response for version 2

**DOI:** 10.3310/nihropenres.14381.r28540

**Published:** 2022-03-28

**Authors:** Hanno Pijl

**Affiliations:** 1 Department of Internal Medicine, Leiden University Medical Center (LUMC), Leiden, The Netherlands

This paper describes the results of a “patient and public involvement activity”, gathering patient (and family members) viewpoints on meal replacement / food-based strategies for weight loss in the treatment of type 2 diabetes (T2D). As most clinical studies evaluating the impact of calorie restriction in T2D were done in Caucasian people, while South Asians are particularly prone to develop the disease *and* constitute a considerable part of the British (and global) population, the study specifically recruited people of South Asian background. 18 people were approached, 13 (of whom only one was male) agreed to participate. 5 of them attended a virtual meeting and the rest came together for a face-to-face gathering. Viewpoints on total diet replacement vs food-based low-calorie diets, acceptability of questionnaires and wearables, as well as (culturally specific) barriers to follow low-energy diets were identified. The authors claim that their results indicate that meal replacement strategies are unacceptable to the South Asian population. Instead, culturally tailored food-based interventions are preferred.  The use of online tools to report diet was presented as unsuitable, while quality of life questionnaires (which ones?) were apparently OK.

 The study design and interpretation of the data have several flaws in my opinion.

1. It seems not appropriate to use the opinion of 13 people, of whom 12 are female, as representative for the South Asian population. Moreover, only 9 of 13 people were actual patients. 4 were family members whose opinion was apparently used to represent the view of relatives/caretakers. The authors fail to scientifically substantiate the generalizability of their conclusions.
2. Unfortunately, the paper does not report the reasons for unacceptability of the meal replacement strategy. It doesn’t even specify the composition of the replacements (it only says “soups and shakes”). In my experience, the taste of meal replacement products is critically important for their acceptability. In addition, social hurdles (not being able to have dinner with friends) are an issue with meal replacement, but this supposedly hinders compliance with food-based strategies as well. Anyway, it seems short-sighted to reject the option of all variants of meal replacement and advocate the use of any food-based strategy on the basis of a single survey evaluating a very limited number (2?, not specified) of options.
3. It is unclear from the list of questions provided in table 1 for how long the patients were supposed to follow dietary prescriptions. And this conceivably matters a lot. For example, using total diet replacement for 1 week every months may be perfectly acceptable for people. Was this an option? Or was dietary intervention primarily meant to induce significant (> 10%) weight loss (as in DiRECT) and therefore very likely to be long-term?
4. The acceptability of questionnaires, e-health tools and online reporting instruments heavily depends on their design and ease of use. The paper does not report which tools were judged by the participants. Eventually, such instruments need to be tested in clinical practice to draw decisive conclusions regarding their usability.

Is the work clearly and accurately presented and does it cite the current literature?

Yes

If applicable, is the statistical analysis and its interpretation appropriate?

Not applicable

Are all the source data underlying the results available to ensure full reproducibility?

No

Is the study design appropriate and is the work technically sound?

No

Are the conclusions drawn adequately supported by the results?

No

Are sufficient details of methods and analysis provided to allow replication by others?

Yes

Reviewer Expertise:

Lifestyle treatment of diabetes and cancer

I confirm that I have read this submission and believe that I have an appropriate level of expertise to state that I do not consider it to be of an acceptable scientific standard, for reasons outlined above.

# Response

**Published:** 2022-04-15

**Authors:** Grace Farhat

**Affiliations:** Manchester Metropolitan University, UK

Thank you very much for giving us the opportunity to revise our manuscript. We have addressed the reviewers’ comments and we feel that this process has significantly improved the quality of our manuscript.

 We very much appreciate the opportunity to improve our manuscript and we hope that it is now suitable for indexing in your journal. Our point-by-point responses are provided in bold below. We look forward to hearing from you.

 This paper describes the results of a “patient and public involvement activity”, gathering patient (and family members) viewpoints on meal replacement / food-based strategies for weight loss in the treatment of type 2 diabetes (T2D). As most clinical studies evaluating the impact of calorie restriction in T2D were done in Caucasian people, while South Asians are particularly prone to develop the disease *and* constitute a considerable part of the British (and global) population, the study specifically recruited people of South Asian background. 18 people were approached, 13 (of whom only one was male) agreed to participate. 5 of them attended a virtual meeting and the rest came together for a face-to-face gathering. Viewpoints on total diet replacement vs food-based low-calorie diets, acceptability of questionnaires and wearables, as well as (culturally specific) barriers to follow low-energy diets were identified. The authors claim that their results indicate that meal replacement strategies are unacceptable to the South Asian population. Instead, culturally tailored food-based interventions are preferred. The use of online tools to report diet was presented as unsuitable, while quality of life questionnaires (which ones?) were apparently OK.

 The study design and interpretation of the data have several flaws in my opinion.

1. It seems not appropriate to use the opinion of 13 people, of whom 12 are female, as representative for the South Asian population. Moreover, only 9 of 13 people were actual patients. 4 were family members whose opinion was apparently used to represent the view of relatives/caretakers. The authors fail to scientifically substantiate the generalizability of their conclusions.

**Author response 1**

**We are very grateful to the reviewer for this comment. Although we tried to ensure that the study cohort was representative of the background population, we accept that the conclusions may be limited by the predominance of women, who historically tend to engage with research more than men. We have highlighted this as a limitation in the “limitations section” of the manuscript. However, we believe that the rich insights gained from our study cohort (coming a more traditional, less represented background) were still useful to help us develop a protocol to test the feasibility of this food-based option before planning a full-scale trial. Moreover, we felt that the inclusion of family members/carers who may be involved in the food preparation have added strength to our PPIE activities.**

1. Unfortunately, the paper does not report the reasons for unacceptability of the meal replacement strategy. It doesn’t even specify the composition of the replacements (it only says “soups and shakes”). In my experience, the taste of meal replacement products is critically important for their acceptability. In addition, social hurdles (not being able to have dinner with friends) are an issue with meal replacement, but this supposedly hinders compliance with food-based strategies as well. Anyway, it seems short-sighted to reject the option of all variants of meal replacement and advocate the use of any food-based strategy on the basis of a single survey evaluating a very limited number (2?, not specified) of options.

**Author response 2**

**Thank you for this comment. In the manuscript, there are added statements from participants indicating their reasons for unacceptability in the section entitled “Views of the use of total diet replacement”. For example, participants explained that they perceived soups as foods that are consumed when people are ill. Another participant mentioned that soups don’t ‘fill you up’ and that other solid foods (such as chapati bread) need to be added.**

**In the extended data (please see** **https://zenodo.org/record/5720754#.YkqowOjMKiw** **), the composition of the soups (including ingredients and flavours) were added to the pack given to participants to read before attending the activities.**

**We would like to make it clear that the results of these activities did not lead for us to completely reject the use of total diet replacement in future trials but, based on the responses obtained, we were encouraged to look at the acceptability of a food-based diet in our planned feasibility study.**

**We have now added this statement to the conclusion section ‘ *This activity*** ***does not negate the use of TDR in this population but provided useful insights to shape a future study looking at the feasibility of food-based intervention for T2D remission in primary care in a high-risk population*’.**

 It is unclear from the list of questions provided in table 1 for how long the patients were supposed to follow dietary prescriptions. And this conceivably matters a lot. For example, using total diet replacement for 1 week every months may be perfectly acceptable for people. Was this an option? Or was dietary intervention primarily meant to induce significant (> 10%) weight loss (as in DiRECT) and therefore very likely to be long-term?

**Author response 3**

**In the extended data (please see** **https://zenodo.org/record/5720754#.YkqowOjMKiw** **), we have indicated to participants in their pack that the low-calorie intervention will last for 12 weeks. This has been also pointed out during the activities. Since we are building on success of the DiRECT trial, we were looking to assess the feasibility of our intervention over 12 weeks (the duration of the DiRECT intervention). Further clarification has now been added to the “Meeting information” section: “The information pack consisted of an example of a diet consisting of soups and shakes to be consumed for 12 weeks”.**

1. The acceptability of questionnaires, e-health tools and online reporting instruments heavily depends on their design and ease of use. The paper does not report which tools were judged by the participants. Eventually, such instruments need to be tested in clinical practice to draw decisive conclusions regarding their usability.

**Author response 4**

**Thank you for the request for further information. The quality-of-life questionnaire** **that we used was the EuroQoL5 EQ-5D-3L which we included in the participant’s pack (** **please see** **https://zenodo.org/record/5720754#.YkqowOjMKiw** **). However, after consideration, we felt that this questionnaire does not add much information and we decided not to use it in our proposed feasibility study. We have also chosen not to report its outcomes in this PPIE paper. The statement reporting acceptability of PPIE has now been removed from the “** **Other components of the intervention” section.**

**As for online reporting instruments, the population comes from a more traditional background (and will be our target cohort in the feasibility study) for whom the use of computers/internet was not acceptable to many, hence we felt it would be more appropriate to use paper-based forms. We have mentioned in the manuscript that 11 participants felt that reporting diet through a phone app would not be suitable.**

# Reviewer report: Reviewer response for version 2

**DOI:** 10.3310/nihropenres.14381.r28497

**Published:** 2022-03-04

**Authors:** Adrian Brown

**Affiliations:** 1 Centre for Obesity Research, University College London, London, UK

Thank you for asking me to re-review this manuscript entitled “Comparing the acceptability of total diet replacement and food-based low-energy diets for type 2 diabetes remission amongst Southeast Asians: a public and patient involvement activity”.

 The authors have systematically addressed the concerns highlighted in the first review, thank you. 

 One minor point is can I please suggest that the authors review the manuscript for spelling and grammar prior to acceptance. E.g., in clinical and research implications, should be ‘test activities’ not ‘tese activities’ and in conclusion: ‘This work’ not ‘his work’.

Is the work clearly and accurately presented and does it cite the current literature?

Yes

If applicable, is the statistical analysis and its interpretation appropriate?

Not applicable

Are all the source data underlying the results available to ensure full reproducibility?

No

Is the study design appropriate and is the work technically sound?

Yes

Are the conclusions drawn adequately supported by the results?

Partly

Are sufficient details of methods and analysis provided to allow replication by others?

Partly

Reviewer Expertise:

Obesity, Type 2 diabetes, Weight Stigma and use of very low and low energy diets in Type 2 diabetes remission and other obesity related diseases.

I confirm that I have read this submission and believe that I have an appropriate level of expertise to confirm that it is of an acceptable scientific standard.

# Reviewer report: Reviewer response for version 1

**DOI:** 10.3310/nihropenres.14349.r28451

**Published:** 2022-01-07

**Authors:** Adrian Brown

**Affiliations:** 1 Centre for Obesity Research, University College London, London, UK

Thank you for asking me to review this manuscript entitled “Comparing the acceptability of total diet replacement and food-based low-calorie diets for type 2 diabetes remission amongst Southeast Asians: a public and patient involvement activity”. This looked at gaining opinions of people from Southeast Asian community regarding what constitutes a culturally acceptable diet.

 This is a very important topic of work with the scarcity of information in the area and with the increased interest of using total diet replacements within clinical practice for remission, the question around whether these types of diets are culturally sensitive and relevant is important. These PPI activities provide novel information suggesting that low energy food-based diets might be preferred within Southeast Asian ethnic groups. This is an interesting report with some important views related to the use of low energy TDR and food-based diets in people from Southeast Asian ethnicity. Including that ready-made catered meals would be welcomed, peer support was key and the need for experienced translators. It was excellent to see the inclusion of a community representative to help with PPI meetings to help with engagement and also translation.

 The major comment within this manuscript is the risk of unconscious researcher bias. Looked at the extended data and the participant information sent prior to the events, the framing of the diets appears different. The information about the TDR diet could be seen as neutral with the following *“People who take part in the diabetes research study will be asked to consume a diet only consisting of soups and shakes for 12 weeks. Food will be reintroduced after then. Here is an example of soups and shakes diets for one day (850 Kcal)”*. However, the food-based diet had the additional of this sentence *“The diet has been tailored to include components of the Mediterranean diet; this diet had many health benefits in the past for preventing diabetes and its complications”*, which appears to the framing the diet more positively. This might have therefore impact on how the participants view the diets and therefore the interpret your data and conclusions. This would benefit from being explored.

 There are a few points that would benefit from being addressed:

- Please can the authors consider changing the terminology from “low calorie” to “low energy”. Although this terminology is used, a calorie is a unit of energy, therefore it should be “low energy diets”.

**Abstract:**

- Please can you change the white population to capital “W”.

**Introduction:**

- When discussing diabetes and risk can you please ensure that you use Type 2 diabetes (T2D) and not diabetes as this could confuse individuals believing other forms can be put into remission e.g., people living with type 2 diabetes not living with diabetes.
- Please can you look at the language that is used as some could be viewed as stigmatising e.g. “tackling” “diabetes crisis” “the burden”. These are combative terms and appears to suggest that people with type 2 diabetes are a burden. Instead, can you look for alternative terms such as “address” instead of “tackling”; “increased diabetes prevalence” instead of “crisis”
- Can you please use the word “remission” instead of “reversed” as T2D cannot be reversed in terms of the underlying biological changes e.g., pancreatic beta cell death, but can be put in remission, transiently, assuming the patient losses weight and keeps it off.
- When discussing the barrier and facilitators to participation in research could the authors, please give an example or two to avoid the reader having to leave the paper completely to look at the reference.

**Methods:**

- Can the authors please explain how the people were selected i.e., how the community education presentative identified people. Also were there only 18 people invited to the PPI group or were there more, in order to get a sense of how many people did not respond to the invitation.

**Meeting information:**

- The Mediterranean diet has also been shown to be able to engender remission from the PREDIMED study – please see my review ^1^ for details. Also, can you reference your comment related to the beneficial effects.

**Data analysis:**

- Please can the authors include more detail on the analysis. Were the interviews transcribed verbatim? Was there then checking of the data and reviewed by another researcher to avoid bias?

**Table 2:**

- Would it be possible to have mean/median for age? Also was this gender or sex, were participants asked about the gender they identified with or was this from medical records? It is possible to identify which ethnic groups the participants were from e.g. Pakistani, Indian as culture is different and help to see generalisability of the data. Also was there data on socio-economic class, this again could impact the interpretation of the data and views.
- Did the participants explain why it was crucial that they adhere to a “strict traditional diet”? This seems a crucial point related to perceptions and an area to address around why people from Southeast Asian ethnic group might prefer using food-based diets and not a TDR. Could these points be addressed to increase acceptance of a TDR.

**Taking part in research:**

- Interesting that your data showed that people hadn’t taken part of research as they hadn’t been asked, please can you check the reference (9) you quote here as it appears not to be related to lack of interest in taking part in research. Instead related to prevalence of type 2 diabetes diagnoses in the UK primary care setting

**Limitations:**

- Only having one researcher review the data could be considered a limitation. If this was not the case, please including this in the methodology.
- It is important that the authors consider the impact of researcher bias and also social desirability bias.

**Clinical and research implications:**

- The conclusion to avoid using TDR completely in this group based on the opinions of 13 people and with the possible chance of unconscious researcher bias could be considered premature.
- With data showing that TDR is effective at engendering remission in individuals from a Middle Eastern population (as you referenced; Taheri *et al.*, 2020 ^2^), that People from Southeast Asia can achieve remission using a liquid low energy diet (Bhatt *et al.*, 2017 ^3^) and qualitative data, although mainly in White individuals, identifying that TDR were easier to follow than initially thought (Harper *et al.*, 2018 ^4^) appears important here. Perceptions of TDR prior to their use are often negative, but opinion frequently changes once they use them. In addition, the team responsible for the DiRECT study in Glasgow are conducting a study in a South Asian population using a TDR which results are expected soon. This will provide data to see if those that used a TDR stuck to it and if it was acceptable.
- It would seem that within this cohort that TDR is not preferred but viewing it as not being an option for remission is questionable considering previous data. It seems that in relation to how PPI might impact on the research, it appears that acceptability should be accessed with through a feasibility trial with a direct comparison between food-based diet and TDR as a start point. While also exploring some of the opinions of the PPI group further to related to why they considered the TDR unacceptable in formal qualitative research.

**Conclusions:**

- In the first sentence should focus on the aim of the study which is remission, it is not clear the reason there is discussion about prevention as this was not the aim of the PPI activities. Please can you review.
- As before please avoid using reverse, please use remission instead.

Is the work clearly and accurately presented and does it cite the current literature?

Yes

If applicable, is the statistical analysis and its interpretation appropriate?

Not applicable

Are all the source data underlying the results available to ensure full reproducibility?

No

Is the study design appropriate and is the work technically sound?

Yes

Are the conclusions drawn adequately supported by the results?

Partly

Are sufficient details of methods and analysis provided to allow replication by others?

Partly

Reviewer Expertise:

Obesity, Type 2 diabetes, Weight Stigma and use of very low and low energy diets in Type 2 diabetes remission and other obesity related diseases.

I confirm that I have read this submission and believe that I have an appropriate level of expertise to confirm that it is of an acceptable scientific standard, however I have significant reservations, as outlined above.

## References

[1] : Dietary strategies for remission of type 2 diabetes: A narrative review. *J Hum Nutr Diet* .2021; 10.1111/jhn.12938 10.1111/jhn.12938 34323335

[2] : Effect of intensive lifestyle intervention on bodyweight and glycaemia in early type 2 diabetes (DIADEM-I): an open-label, parallel-group, randomised controlled trial. *The Lancet Diabetes & Endocrinology* .2020;8(6) : 10.1016/S2213-8587(20)30117-0 477-489 10.1016/S2213-8587(20)30117-0 32445735

[3] : Effect of a Low-Calorie Diet on Restoration of Normoglycemia in Obese subjects with Type 2 Diabetes. *Indian J Endocrinol Metab* .21(5) : 10.4103/ijem.IJEM_206_17 776-780 10.4103/ijem.IJEM_206_17 28989891PMC5628553

[4] : Experiences of using very low energy diets for weight loss by people with overweight or obesity: a review of qualitative research. *Obes Rev* .19(10) : 10.1111/obr.12715 1412-1423 10.1111/obr.12715 30144269

# Response

**Published:** 2022-02-11

**Authors:** Grace Farhat

**Affiliations:** Manchester Metropolitan University, UK

Thank you for asking me to review this manuscript entitled “Comparing the acceptability of total diet replacement and food-based low-calorie diets for type 2 diabetes remission amongst Southeast Asians: a public and patient involvement activity”. This looked at gaining opinions of people from Southeast Asian community regarding what constitutes a culturally acceptable diet.

***Author response*** **:**

**Thank you for taking the time to thoroughly review our manuscript and for providing helpful insights.**

 This is a very important topic of work with the scarcity of information in the area and with the increased interest of using total diet replacements within clinical practice for remission, the question around whether these types of diets are culturally sensitive and relevant is important. These PPI activities provide novel information suggesting that low energy food-based diets might be preferred within Southeast Asian ethnic groups. This is an interesting report with some important views related to the use of low energy TDR and food-based diets in people from Southeast Asian ethnicity. Including that ready-made catered meals would be welcomed, peer support was key and the need for experienced translators. It was excellent to see the inclusion of a community representative to help with PPI meetings to help with engagement and also translation.

 The major comment within this manuscript is the risk of unconscious researcher bias. Looked at the extended data and the participant information sent prior to the events, the framing of the diets appears different. The information about the TDR diet could be seen as neutral with the following *“People who take part in the diabetes research study will be asked to consume a diet only consisting of soups and shakes for 12 weeks. Food will be reintroduced after then. Here is an example of soups and shakes diets for one day (850 Kcal)”*. However, the food-based diet had the additional of this sentence *“The diet has been tailored to include components of the Mediterranean diet; this diet had many health benefits in the past for preventing diabetes and its complications”*, which appears to the framing the diet more positively. This might have therefore impact on how the participants view the diets and therefore the interpret your data and conclusions. This would benefit from being explored.

***Author response*** **:**

**This is a good point. We primarily aimed to look at the acceptability of TDR versus food-based diet, with a Mediterranean component because of its established health benefits in the south Asian population.**

**The sentence that acknowledged the benefits of a Mediterranean diet (in the document provided to participants) was mainly to provide a justification to why we are including a Mediterranean component in an Asian diet, as it could be unacceptable and unusual to some. Although this has made the food-based diet look more positive, we don’t believe this has impacted the opinion of our panel.**

**All discussions focused on the culturally appropriate elements in the food-based diet (spices, traditional foods etc…) that made it appealing to this group. Mediterranean diet was only brought up during the discussion when we asked about its acceptability.**

**This point has been added to the Results section of the manuscript which now reads, “ *Although including a Mediterranean component in the food-based diet (together with its potential beneficial effects) might have made the food-based diet appear more positive, this particular element was not the subject of discussion in both activities. All panel discussions focused on the culturally appropriate elements in the food-based diet such as spices and traditional foods, that made it more appealing*.”**

**However, response to this helpful comment, we have added the following sentence to the limitation section of the discussion, “ *Our description of the potential health benefits of a Mediterranean diet may have positively impacted how participants viewed the food-based diet*.”**

 There are a few points that would benefit from being addressed:

 Please can the authors consider changing the terminology from “low calorie” to “low energy”. Although this terminology is used, a calorie is a unit of energy, therefore it should be “low energy diets”.

***Author response*** **:**

**We agree and this terminology has been changed throughout the manuscript.**

 Abstract: Please can you change the white population to capital “W **”.**

***Author response*** **:**

**As per government guidelines,** **the “preferred style is not to capitalise ethnic groups, (such as ‘black’ or ‘white’) unless that group’s name includes a geographic place (for example, ’Asian’, ‘Indian’ or ‘black Caribbean’)”. Please see** Writing about ethnicity - GOV.UK (ethnicity-facts-figures.service.gov.uk)

 Introduction: When discussing diabetes and risk can you please ensure that you use Type 2 diabetes (T2D) and not diabetes as this could confuse individuals believing other forms can be put into remission e.g., people living with type 2 diabetes not living with diabetes.

***Author response*** **:**

**Thank you. These statements have been amended.**

 Please can you look at the language that is used as some could be viewed as stigmatising e.g. “tackling” “diabetes crisis” “the burden”. These are combative terms and appears to suggest that people with type 2 diabetes are a burden. Instead, can you look for alternative terms such as “address” instead of “tackling”; “increased diabetes prevalence” instead of “crisis”

***Author response*** **:**

**These terms have been amended and alternative language has been used.**

 Can you please use the word “remission” instead of “reversed” as T2D cannot be reversed in terms of the underlying biological changes e.g., pancreatic beta cell death, but can be put in remission, transiently, assuming the patient losses weight and keeps it off.

***Author response*** **:**

**We are grateful for this comment. The term “reversed” has been replaced with “remission”.** 

 When discussing the barrier and facilitators to participation in research could the authors, please give an example or two to avoid the reader having to leave the paper completely to look at the reference.

***Author response*** **:**

**Examples of barriers and facilitators to participation have been added to the introduction which reads, “ *The barriers and facilitators to participation in health and T2D diabetes research within the south Asian population (such as perceived participation to improve health, cultural and language barriers, and lack of interest) have been described elsewhere ^16^. It is therefore important to look at the suitability and barriers for success for low energy interventions as a means of inducing T2D remission in this population.”***

 Methods: Can the authors please explain how the people were selected i.e., how the community education presentative identified people. Also were there only 18 people invited to the PPI group or were there more, in order to get a sense of how many people did not respond to the invitation.

***Author response*** **:**

**The community representative recruited participants by getting in touch with a local GP practice and through word-of mouth in community local groups. This has resulted in 18 people being approached and 13 responding. We were only aiming to recruit around 12 people.**

**We have made this information clearer in the manuscript which now reads, “ *Patients and family members were recruited face-to-face and by telephone through a GP practice and with the assistance of a community education representative with strong community links helping to spread the word within different sub-ethnic populations (Pakistani, Bangladeshi and Indian groups) in community local groups. Invitations included the researchers’ contact details and were sent out by email and “Whatsapp” application either by the researcher directly or through the community representative. Overall, 18 people were approached, and 13 people accepted the invitation. Inclusion criteria included men and women over 18 years of age from a south Asian background who are either patients with type 2 diabetes or have someone with type 2 diabetes in the household. English and non-English speakers were invited to attend, and the community representative was available to help with the translation*.”**

 Meeting information: The Mediterranean diet has also been shown to be able to engender remission from the PREDIMED study – please see my review ^1^ for details. Also, can you reference your comment related to the beneficial effects.

***Author response*** **:**

**Thank you. We have referenced a study linking Mediterranean diet to T2D remission & beneficial effects on cardiovascular health in the introduction section “ *The information pack consisted of an example of a diet consisting of soups and shakes, a 3-day low energy food-based diet plan [they were provided with information to explain that the diet has Mediterranean components (olive oil, fruits and vegetables) which have beneficial effects on remission of T2D and cardiovascular health“.***

 Data analysis: Please can the authors include more detail on the analysis. Were the interviews transcribed verbatim? Was there then checking of the data and reviewed by another researcher to avoid bias?

***Author response*** **:**

**The interviews were first transcribed verbatim and then a summary of important points was developed. This summary has been reviewed by the community representative. This sentence is now added to the “data analysis” section which reads, “ *Interviews were first transcribed verbatim by the researcher (GF), and a detailed summary of all responses was then produced. This summary was reviewed by the community representative. Relevant information was retained and included in the report.”***

**The data was not checked by another researcher; a point that has been included as a study limitation. The relevant text in the discussion reads, “ *Our data could have benefited by being reviewed by more than one researcher to reduce the potential for researcher bias”.***

 Table 2: Would it be possible to have mean/median for age? Also was this gender or sex, were participants asked about the gender they identified with or was this from medical records? It is possible to identify which ethnic groups the participants were from e.g. Pakistani, Indian as culture is different and help to see generalisability of the data. Also was there data on socio-economic class, this again could impact the interpretation of the data and views.

***Author response*** **:**

**Thanks for asking these points for clarification. We recorded participants by their age category and not by their numeric age. Therefore, we are not able to provide the mean and median ages; just the median age category (40-64 years).**

**Gender is how our participants identified themselves.**

**Information on sub-ethnicity and socio-economic class has been added to the manuscript. Please see the revised Table 2.**

 Did the participants explain why it was crucial that they adhere to a “strict traditional diet”? This seems a crucial point related to perceptions and an area to address around why people from Southeast Asian ethnic group might prefer using food-based diets and not a TDR. Could these points be addressed to increase acceptance of a TDR.

***Author response*** **:**

**We understood this is mostly related to the** **association between diet and cultural identity** **which has been also backed by the literature ^1^. Participants mentioned that this is linked to their culture (this statement is now added to the results section), and this aspect is particularly important for those who were born outside the UK. Those born in the UK were less keen about a strict traditional diet.**

**The results section now reads, “ *For those born outside the UK (n=8), it was reported that it would be crucial that they adhere to a strict traditional diet as this is linked to their home culture, while south Asians born in the UK were more willing to accept non-traditional foods".***

***^1^** Diabetes UK (2009). Diabetes UK and South Asian Health Foundation recommendations on diabetes research priorities for British South Asians. Available at https://www.diabetes.org.uk/resources-s3/2017-11/south_asian_report.pdf*

 Taking part in research: Interesting that your data showed that people hadn’t taken part of research as they hadn’t been asked, please can you check the reference (9) you quote here as it appears not to be related to lack of interest in taking part in research. Instead related to prevalence of type 2 diabetes diagnoses in the UK primary care setting

***Author response*** **:**

**Thanks for pointing out this error and we have found an alternative study that is in line with our outcomes. We have changed this reference to, *“* *This statement is in line with the findings of a previous report showing that people from this population did not participate in research studies because they have never been asked".***

 Limitations: Only having one researcher review the data could be considered a limitation. If this was not the case, please including this in the methodology.

 It is important that the authors consider the impact of researcher bias and also social desirability bias.

***Author response*** **:**

**These limitations have been included to the limitations section of the discussion which now reads, “ *Our data could have benefited by being reviewed by more than one researcher to reduce potential researcher bias. There may also have been social desirability bias amongst the PPIE group”.***

 Clinical and research implications: The conclusion to avoid using TDR completely in this group based on the opinions of 13 people and with the possible chance of unconscious researcher bias could be considered premature.

 With data showing that TDR is effective at engendering remission in individuals from a Middle Eastern population (as you referenced; Taheri *et al.*, 2020 ^2^ ), that People from Southeast Asia can achieve remission using a liquid low energy diet (Bhatt *et al.*, 2017 ^3^ ) and qualitative data, although mainly in White individuals, identifying that TDR were easier to follow than initially thought (Harper *et al.*, 2018 ^4^ ) appears important here. Perceptions of TDR prior to their use are often negative, but opinion frequently changes once they use them. In addition, the team responsible for the DiRECT study in Glasgow are conducting a study in a South Asian population using a TDR which results are expected soon. This will provide data to see if those that used a TDR stuck to it and if it was acceptable.

 It would seem that within this cohort that TDR is not preferred but viewing it as not being an option for remission is questionable considering previous data. It seems that in relation to how PPI might impact on the research, it appears that acceptability should be accessed with through a feasibility trial with a direct comparison between food-based diet and TDR as a start point. While also exploring some of the opinions of the PPI group further to related to why they considered the TDR unacceptable in formal qualitative research.

***Author response*** **:**

**Thank you for these helpful insights. We have been considering these points in the planning of a feasibility study. There has been further research showing acceptability of TDR among south Asians which has raised many questions in relation to why outcomes of this PPIE activity have been different. It may be possible that the different sub-ethnic groups and the more traditional populations might be less accepting of TDR. Additionally, in view of the perceived link between food and cultural identity in the south Asian population, looking for alternative options to TDR might encourage more people to take part in studies. We are aiming to develop a single arm feasibility study looking at the acceptability of food-based catered meals diet in the south Asian population, which would lay the ground for a full intervention comparing different diet options.**

**In response to these helpful comments, we have changed the first sentence of the Clinical and research implications section so that it reads, “ *Data from this cohort, including participants from a more traditional south Asian sub-group, suggest that TDR may have limited acceptability in this patient population. *We have also included the following statements in the limitation sections “ *Lastly, the lack of knowledge and use of TDR might have affected their acceptability. White individuals have previously expressed negative perceptions of TDR too, yet their opinions changed after use ^23^. Future studies will be able to identify whether this will be the case in the south Asian population”*.**

 Conclusions: In the first sentence should focus on the aim of the study which is remission, it is not clear the reason there is discussion about prevention as this was not the aim of the PPI activities. Please can you review.

***Author response*** **:**

**The first sentence of the conclusion has been amended so that it reads, “ *The south Asian population is an important target group for interventions designed to induce T2D remission.”***

 As before please avoid using reverse, please use remission instead.

***Author response*** **:**

**We have amended these statements accordingly.**

# Reviewer report: Reviewer response for version 1

**DOI:** 10.3310/nihropenres.14349.r28457

**Published:** 2021-12-13

**Authors:** Alisha N. Wade, Zodwa Dire

**Affiliations:** 1 MRC/Wits Rural Public Health and Health Transitions Research Unit (Agincourt), School of Public Health, Faculty of Health Sciences, University of the Witwatersrand, Johannesburg, South Africa; 2 Department of Internal Medicine, University of the Witwatersrand, Johannesburg, South Africa

The authors present a comparison between the acceptability of total diet replacement and food-based low calorie diets for type 2 diabetes remission in individuals of Southeast Asian ethnicity in the UK. Our major concern is whether this manuscript reports patient and public involvement as the authors assert or whether it is in fact qualitative formative work.

Major comments

 1. As noted above, the key question is whether this manuscript details patient and public involvement or qualitative formative work. If it is the latter, ethical approval would have been required for the work and given that this was not done, the manuscript cannot be approved. Having reviewed guidance that highlights the differences between the two, we believe this constitutes qualitative research based on the following:

- Participants are primarily providing their perspectives on the acceptability of the method of consuming low calorie diets. There is no evidence of active discussion in which both researchers and participants offer views and participants contribute to decisions on the research question, design etc.
- Although feedback was sought on a proposed questionnaire and the use of step counters, it appeared rather superficial and there was no evidence as to how this feedback modified the researchers' plans.
- The participants appeared to have no prior experience of research while those in patient and public involvement usually have some experience of research, either as part of a charity or lay group.
- The questions put to participants were quite focused on a particular experience.

If it is accepted that this is indeed qualitative research, then apart from the absence of ethical approval, there are other issues to be addressed before the article is suitable for indexing.

- The authors need to provide more data on the context from which participants were recruited.
- There needs to be greater clarity about how participants were recruited-the information given appears to be contradictory.
- How was the number of people invited decided?
- The method of data analysis needs to be properly reported.
- The results need to be reported using standard qualitative reporting format. It would also be useful to include the diabetes status when reporting characteristics of the sources of quotes.
- It is less useful to report quantitative data as the numbers are too small to draw any conclusions.
- The authors should reflect on the influence of social desirability bias on their findings.

Minor comments

 1. The introduction would benefit from being more focused.

 2. The transcripts are not available and the analysis therefore not repeatable.

 3. The entire article needs to be carefully proof read.

Is the work clearly and accurately presented and does it cite the current literature?

Partly

If applicable, is the statistical analysis and its interpretation appropriate?

Not applicable

Are all the source data underlying the results available to ensure full reproducibility?

No

Is the study design appropriate and is the work technically sound?

Partly

Are the conclusions drawn adequately supported by the results?

Partly

Are sufficient details of methods and analysis provided to allow replication by others?

No

Reviewer Expertise:

Endocrinology and metabolism in lower and middle income countries

We confirm that we have read this submission and believe that we have an appropriate level of expertise to state that we do not consider it to be of an acceptable scientific standard, for reasons outlined above.

# Response

**Published:** 2022-02-11

**Authors:** Grace Farhat

**Affiliations:** Manchester Metropolitan University, UK

The authors present a comparison between the acceptability of total diet replacement and food-based low calorie diets for type 2 diabetes remission in individuals of Southeast Asian ethnicity in the UK. Our major concern is whether this manuscript reports patient and public involvement as the authors assert or whether it is in fact qualitative formative work.

Major comments

 1. As noted above, the key question is whether this manuscript details patient and public involvement or qualitative formative work. If it is the latter, ethical approval would have been required for the work and given that this was not done, the manuscript cannot be approved.

 Having reviewed guidance that highlights the differences between the two, we believe this constitutes qualitative research based on the following: Participants are primarily providing their perspectives on the acceptability of the method of consuming low calorie diets. There is no evidence of active discussion in which both researchers and participants offer views and participants contribute to decisions on the research question, design etc.

***Author response*** **:**

**We explain below why we strongly believe that our work qualifies as a PPIE activity and not as qualitative work.** **We have received consent from participants to publish these PPIE views anonymously, therefore the work has entirely appropriate ethical standards.**

**The reviewers provide useful** guidance **that clarifies the difference between PPIE activities and qualitative research. Here we provide evidence that our work is a PPIE activity based on the seven characteristics that differentiate PPIE from qualitative research (see here).**

**Research question: Our stated aim was to understand the views of people from the south Asian population on potential dietary approaches to achieve diabetes remission. Our research was not addressing a single research question (qualitative research) but rather the PPIE group helped us to select and refine the research question for our future clinical trial. For example, in our discussions with our participants, we were rather surprised that they expressed** a **preference for a culturally tailored low energy food-based diet delivered as catered meals. Participants suggested that catered meals would increase adherence and knowledge in portion size and cooking methods. Therefore, this has led us to design a completely different dietary intervention than the one that we originally thought might be acceptable.**

1. **Practical approach: our approach to gathering information was arranged in a way that suited both researchers and participants (PPIE activity) and did not follow any method based on theory (qualitative research).**
2. **People involved: our research is focused on people from the south Asian population but apart from this criterion we did not select participants in other ways which make this a PPIE activity rather than qualitative research.**
3. **Ethical approval: this was not sought because we firmly believe that this is PPIE activity and not qualitative research based on these criteria.**
4. **People’s input: as we have described in the paper, we used people’s input to help us design and undertake our future research. For example, based on the rather unexpected views of our participants we have designed our next study to assesses the feasibility and acceptability of a culturally appropriate food-based low energy intervention delivered through catered meals. We have not used people’s input simply as data to address a specific research question. This makes this a PPIE activity rather than qualitative research.**
5. **Power: the views of our participants completely changed our thoughts about the nature of an appropriate future intervention in this patient population. As mentioned above, the research participants guided us in designing a more appropriate clinical intervention which will be tested in a clinical trial therefore this is clearly a PPIE activity rather than qualitative research in which only the researchers have the power to influence the conduct of the research.**
6. **Use of the findings: we recognise that the opinions of our participants relate to them and that their views may have limited generalisability. For example, in the clinical implications section of the manuscript we write, “ *Data from this cohort, including participants from a more traditional south Asian sub-group, suggest that TDR may have limited acceptability in this patient population*.” We also write in the limitations section, “ *Whilst attempts were made to ensure that the study cohort was representative of the background population, the small number of participants and our recruitment methods could have impacted the conclusions drawn from these meetings*.” We recognise that we need to study a larger sample that may be more representative of the SAP and therefore our feasibility study will recruit a larger cohort from a wider geographical region. Since we openly recognise the limited generalisability of our findings, this makes this a PPIE activity rather than qualitative research.**

**Therefore, based on the criteria above, we conclude that our study clearly represents a PPIE activity rather than a qualitative research study.**

 Although feedback was sought on a proposed questionnaire and the use of step counters, it appeared rather superficial and there was no evidence as to how this feedback modified the researchers' plans.

***Author response*** **:**

**The “Clinical and research implications” and conclusion sections in the manuscript have indicated how this feedback has helped in our research plans with the following statements:**

**“Data from this cohort, including participants from a more traditional south Asian sub-group, suggest that TDR may have limited acceptability in this patient population. This work will help us design a randomised controlled study using low energy diets in South Asian people with T2D with the aim of inducing remission. These activities suggest the potential utility of a food-based low energy intervention, including looking at the feasibility of administering catered meals in primary care when compared to usual care. Meal plans will be prepared together with members of the community and patient support members. An education element to increase knowledge of T2D risk and healthy eating will be considered. Through the** **Greater Manchester Strategic Clinical Network** **and the** **Research for the Future** **intervention, we will promote engagement with this research for people with diabetes from the south Asian population."**

**“The south Asian population is an important target group for interventions designed to induce T2D remission. This activity provided useful insights to shape a future study looking at the feasibility of food-based intervention for T2D remission in primary care in a high-risk population. It will encourage more patients to become involved in T2D research, which may lead in the long-term to improved quality of life, health, and economic benefits**”.

 The participants appeared to have no prior experience of research while those in patient and public involvement usually have some experience of research, either as part of a charity or lay group.

***Author response*** **:**

**Whilst we agree that it would be ideal to include people with prior research experience, it is not often possible especially in such an underrepresented group. As we have mentioned above, we did not select participants based on the prior research experience.**

 The questions put to participants were quite focused on a particular experience.

***Author response:***

**Whilst researchers had broadly set the agenda, the session was flexible enough to allow for public-led items or a change of direction.**

 If it is accepted that this is indeed qualitative research, then apart from the absence of ethical approval, there are other issues to be addressed before the article is suitable for indexing. 

***Author response:***

**In view of the evidence that this is a PPIE activity, the points raised below would be more relevant for qualitative research than a PPIE, but if the reviewer and Editorial team feel that additional information is required in the context of a PPIE activity then we will be happy to respond.**

 The authors should reflect on the influence of social desirability bias on their findings.

***Author response*** **:**

**We have included social desirability as a limitation in the “limitations section”.**

Minor comments

 1. The introduction would benefit from being more focused.

***Author response*** **:**

**We have modified the introduction to make this more focussed (please see the revised manuscript).**

 2. The transcripts are not available and the analysis therefore not repeatable.

***Author response*** **:**

**We did not request participants’ consent to publish their transcripts and have added this as a limitation.**

 3. The entire article needs to be carefully proofread.

***Author response*** **:**

**The article has been reviewed by all authors and proofread by the journal.**
